# A fully human anti-IL-7Rα antibody promotes antitumor activity against T-cell acute lymphoblastic leukemia

**DOI:** 10.1038/s41375-019-0434-8

**Published:** 2019-03-08

**Authors:** Padma Akkapeddi, Rita Fragoso, Julie A. Hixon, Ana Sofia Ramalho, Mariana L. Oliveira, Tânia Carvalho, Andreas Gloger, Mattia Matasci, Francisco Corzana, Scott K. Durum, Dario Neri, Gonçalo J. L. Bernardes, João T. Barata

**Affiliations:** 1 Instituto de Medicina Molecular João Lobo Antunes, Faculdade de Medicina da Universidade de Lisboa, Av. Prof. Egas Moniz, 1649-028 Lisboa, Portugal; 2 Cytokines and Immunity Section, Center for Cancer Research, National Cancer Institute, Frederick, MD USA; 3 Departamento de Química, Universidad de La Rioja, Centro de Investigación en Síntesis Química, 26006 Logroño, Spain; 4 Philochem AG, Libernstrasse 3, Otelfingen, Switzerland; 5 Department of Chemistry and Applied Biosciences, Swiss Federal Institute of Technology (ETH Zürich), Vladimir-Prelog-Weg 4, Zürich, Switzerland; 6 Department of Chemistry, University of Cambridge, Lensfield Road, CB2 1EW Cambridge, UK

**Keywords:** Targeted therapies, Acute lymphocytic leukaemia

## Abstract

T-cell acute lymphoblastic leukemia (T-ALL) is an aggressive hematological cancer for which treatment options often result in incomplete therapeutic efficacy and long-term side-effects. Interleukin 7 (IL-7) and its receptor IL-7Rα promote T-ALL development and mutational activation of IL-7Rα associates with very high risk in relapsed disease. Using combinatorial phage-display libraries and antibody reformatting, we generated a fully human IgG1 monoclonal antibody (named B12) against both wild-type and mutant human IL-7Rα, predicted to form a stable complex with IL-7Rα at a different site from IL-7. B12 impairs IL-7/IL-7R-mediated signaling, sensitizes T-ALL cells to treatment with dexamethasone and can induce cell death per se. The antibody also promotes antibody-dependent natural killer-mediated leukemia cytotoxicity in vitro and delays T-cell leukemia development in vivo, reducing tumor burden and promoting mouse survival. B12 is rapidly internalized and traffics to the lysosome, rendering it an attractive vehicle for targeted intracellular delivery of cytotoxic cargo. Consequently, we engineered a B12–MMAE antibody–drug conjugate and provide proof-of-concept evidence that it has increased leukemia cell killing abilities as compared with the naked antibody. Our studies serve as a stepping stone for the development of novel targeted therapies in T-ALL and other diseases where IL-7Rα has a pathological role.

## Introduction

T-cell acute lymphoblastic leukemia (T-ALL) is an aggressive hematological cancer that results from the transformation of thymic T-cell precursors, accounting for 10–15% of pediatric, and 20–25% of adult, ALL cases. Although treatment outcome has dramatically improved throughout the years, this success has been achieved using intensive chemotherapy that often leads to long-term severe side-effects. Moreover, more than half of the adult, and up to 30% of childhood, T-ALL cases relapse [1]. Thus, there is a pressing need for the development of more selective, safer, and efficient treatments.

Interleukin 7 (IL-7), a cytokine produced in the thymus, bone marrow, and other tissues, and its receptor (IL-7R, a heterodimer of IL-7Rα and γc) are essential for normal lymphoid development [2]. However, the IL-7/ IL-7R axis can also play a significant role in leukemogenesis. High IL-7R expression in murine thymocytes associates with proliferation and leukemogenesis [3], and IL-7 can act as a lymphoid oncogene in the mouse [4, 5]. In agreement, IL-7 accelerates human T-ALL development in vivo [6]. Moreover, studies in vitro showed that leukemia blasts from a majority of the patients (~ 70%) display IL-7R and that IL-7 promotes their survival and proliferation [5, 7–9]. Notably, ~ 10% of pediatric T-ALL patients display IL-7Rα gain-of-function mutations, which lead to constitutive activation of downstream signaling and subsequent promotion of cell transformation and tumorigenesis [10], associating with very poor prognosis in relapsed patients [11]. At the molecular level, IL-7 stimulation and IL-7R mutational activation promote T-ALL cell cycle progression and viability by activating oncogenic pathways such as PI3K/Akt/mTOR and JAK/STAT [12–14]. In addition, IL-7 stimulation of T-ALL cells interferes with drug-induced apoptosis and cell cycle arrest induced by rapamycin [15], prednisolone [16], dexamethasone, and doxorubicin [17], whereas mutant IL-7R-mediated signaling contributes to resistance to glucocorticoids [18]. Overall, these findings indicate that biopharmaceuticals targeting the IL-7/IL-7Rα axis may benefit a large fraction of T-ALL patients (70–80% of the cases), including those with ultra-high risk relapsed disease.

Antibody therapies have become the strategy of choice for the treatment of various forms of cancer [19, 20]. Several antibody products have received marketing authorization for the treatment of hematological malignancies, including against CD20, CD38, and CD52. The approval of brentuximab vedotin (an antibody–drug conjugate against CD30) for the treatment of certain forms of Hodgkin's lymphoma and anaplastic large cell lymphoma, and of blinatumomab (a bispecific antibody recognizing CD19 and CD3) for philadelphia-negative relapsed/refractory ALL, confirms a growing interest in antibody-based therapies to treat blood cancers [21]. Fully human monoclonal antibodies are particularly attractive, as these products should display reduced immunogenicity in patients as compared with antibodies of rodent origin.

Here, we developed and characterized a fully human monoclonal antibody against IL-7Rα, providing clear proof-of-concept for the development of antibody-based treatments for T-ALL and other cancers relying on IL-7/IL-7R-mediated signals.

## Materials and methods

### Phage-display and antibody selection

Biotinylated IL-7Rα-ECD was immobilized onto Streptavidin-coated strips (Roche) at a final concentration of 250 nm in phosphate-buffered saline (PBS). Selections were performed with two Phage-Display libraries, namely ETH2Gold and PhiloDiamond [22, 23], following an established protocol [22]. Soluble antibody fragments were detected using enzyme-linked immunosorbent assay after several rounds of panning. Selected single-chain variable fragments (scFv) were Sanger-sequenced and clones with intact sequence were expressed in large scale in *Escherichia coli* TG1 strain.

### Antibody cloning, expression, and characterization

scFv antibodies were expressed in the TG1 strain with IPTG induction at 30 °C and purified from the bacterial supernatant by affinity chromatography using Protein A-Sepharose (Sino Biological) followed by size-exclusion chromatography (ÄKTA FPLC GE Healthcare, Superdex 75 column). The scFv fragment of clone B12 was reformatted to IgG1 by step-wise cloning giving rise to pMM137-IgG(B12). IgG(B12) was routinely expressed in suspension-adapted mammalian Chinese Hamster Ovary (CHO) cells (Invitrogen) and purified using protein A-sepharose and size-exclusion chromatography (Superdex 200 column), as previously described [24].

### Surface plasmon resonance spectroscopy

Affinity measurements of the purified antibody fragments were performed by surface plasmon resonance spectroscopy using BIAcore 3000 (BIAcore AB). Binding kinetics was analyzed in real-time on CM5 microsensor chip, coated with IL-7Rα recombinant protein, resulting in 3000 response units of coating. The reference flow was left uncoated to facilitate background subtraction. Freshly prepared monomeric fractions of scFv(B12) and IgG(B12) were used immediately after size-exclusion chromatography for BIAcore analysis, to minimize avidity effects from diabody or aggregate formation. Binding constants were calculated using BIAevaluation software (version 3.2).

### Generation of IL-7Rα antibody–drug conjugate

The purified Ig(B12) was conjugated with MMAE using a protease-cleavable valine–citruline linker with a carbonyl acrylic acid head-group. In brief, interchain disulfide bonds of a full-length IgG(B12) were reduced using excess amounts of DTT and reacted with 60 equivalents of carbonyl acrylic acid–valine–citruline–MMAE molecule [25]. The reaction was stirred at 37 °C for 8–10 h. The antibody–drug conjugate was then purified using a desalting column and concentrated using Vivaspin devices (GE Healthcare). The average drug-to-antibody ratio was 4:1 as determined by native mass spectrometry.

### 3D modeling of B12-IL-7Rα interaction

A homology model of B12 antibody was generated through SWISS-MODEL platform [26]. Then, 0.5 µs molecular dynamics (MD) simulations were run to obtain an equilibrated structure, as described in the Supplementary Methods. Docking calculations were performed with PatchDock Server and FireDock [27] between B12 and unglycosylated human IL-7Rα (IL-7Rα, pdb ID: 3DI2) to obtain the complex structure. The best solutions are shown in Table S1. The solution with the lowest binding energy was then subjected to 0.5 µs MD simulations.

### Cells

Primary human T-ALL cells were collected from peripheral blood or bone marrow of patients at diagnosis [28]. We established the primary-like IL-7-dependent cell line TAIL7 [29]. Human TAIL7, HPB-ALL, DND4.1, MOLT4, TALL1, and Jurkat cell lines, and murine Ba/F3 and D1 cell lines transduced with mutant or wild-type IL-7R, were routinely cultured at 37 °C with 5% CO_2_ in RPMI-1640 medium supplemented with 10% (vol/vol) fetal bovine serum and 2 mm l-glutamine, plus IL-7 when necessary. Primary T-ALL cells and normal human thymocytes were obtained after informed consent and Institutional Review Board approval. Culture experiments were performed with or without 50 ng/ml recombinant human IL-7 (Peprotech).

### Flow cytometry

Binding of B12 to native IL-7Rα expressed at the surface of indicated cells was analyzed by flow cytometry. Cells were incubated with B12 or isotype control at 4 °C for 30 min, washed with ice-cold PBS and the primary antibody detected using Goat alexa 647-conjugated anti-human (H + L) antibody (ThermoFischer Scientific). Cell viability was determined by forward scatter/side scatter distribution, and by Annexin V (eBioscience) and 7AAD (BD Bioscience) staining. Samples were acquired using FACS Fortessa I/II (BD Bioscience) and analyzed using FlowJo (Tree Star).

### Immunoblotting

After the indicated conditions and time points, cell lysates were prepared and equal amounts of protein (50 µg/sample) were analyzed by 12% sodium dodecyl sulfate polyacrylamide gel electrophoresis (SDS-PAGE), transferred onto nitrocellulose membranes, and immunoblotted with the indicated antibodies: pSTAT5 (Y694/Y699) (Millipore), pAKT (S473), pERK1/2 (T202/Y204), pS6 (S235/S236) (Cell Signaling Technology), and actin (Santa Cruz Biotechnology). Detection was performed by incubation with horseradish peroxidase-conjugated anti-mouse (Promega), anti-rabbit (Promega), or anti-goat (Santa Cruz Biotechnology) immunoglobulin (IgG; 1:5000 dilution), as appropriate and developed by chemiluminescence.

### ADCC in vitro assay

Antibody-dependent cell-mediated cytotoxicity (ADCC) activity was determined by measuring lactate dehydrogenase (LDH) release using Cytotoxicity Detection KitPLUS (Roche). In brief, T-ALL patient-derived xenograft (PDX) target cells (10^4^) were incubated for 30 min with 10 μg/ml B12 or recombinant human IgG1 Fc (BioX) as isotype control. Human NK effector cells isolated from healthy donors were added at the indicated effector-to-target (E:T) ratio. After 4 h of incubation, reaction mixture was added to the samples and LDH release measured using a Molecular Devices VersaMax ELISA reader. The percentage of cytotoxicity was determined as percent lysis = (effector + target mix−effector cell alone)−target cell alone/lysed targets-targets alone ×100.

### Confocal microscopy

Antibody colocalization studies were performed by confocal microscopy analysis in HPB-ALL cells, as previously described [30]. In brief, cells were incubated with B12 for 30–45 min, at 4 °C, washed, re-suspended in PBS and incubated with secondary anti-human IgG (H + L)-Alexa 647 antibody (Invitrogen) for 30 min at 4 °C. Cells were then carefully washed with ice-cold PBS and stimulated with or without 50 ng/ml IL-7 for 30 min at 37 °C. Cells were washed, fixed with ice-cold methanol for 2–3 min at − 20 °C, permeabilized and washed with PBS plus 0.05% tween, and incubated for 1 h at room temperature in permeabilization buffer with antibodies against: clathrin heavy-chain (X22-Alexa Fluor 555), EEA-1 (EPR4245-Alexa-647) and LAMP-2 (Alexa Fluor 555; all from ThermoFisher Scientific), followed by 4′,6-diamidino-2-phenylindole nuclear staining. Image acquisition was performed with the pinhole aperture set to 1 Airy Unit for the highest wavelength (633 nm) and adjusted for the lower wavelengths to maintain the same optical slice thickness for all channels. Up to 10 different fields of view with ~80 cells per field of view were collected for quantification. Percent c-localization was determined by counting the fraction of cells showing at least one colocalization event (minimum 3 × 3 pixels) between fluorophores from cells with intact nuclei and presenting both fluorophores.

### In vivo studies with D1 IL-7Rα mutant-expressing leukemia T cells

All protocols and animal procedures were approved by institutional Animal Ethics committee of Instituto de Medicina Molecular and followed the recommendations for care and use of laboratory animals by European commission and Portuguese authorities. Rag1−/− mice were obtained from Charles River Laboratories. All mice were 8–12 weeks old at the start of experiments. Green fluorescent protein (GFP positive D1mutP2 cells (5 × 10^6^) were injected intravenously in the tail (200 μl per injection) into Rag1−/− mice. Antibody treatment (250 μg/injection) started either 1 or 7 days post engraftment, at the indicated frequency. Mice were either analyzed for survival or killed on day 15 for comparison of leukemia burden, in which case organs were collected for post-mortem analysis by flow cytometry and histology, as described [31]. In some cases, to identify NK and macrophages, respectively, organ single-cell suspensions were stained with antibodies against CD56 or CD11b (eBiosciences). Tumor burden was determined by the frequency of GFP-positive cells in the different organs. All the samples were acquired and analyzed using FACS Fortessa (Becton Dickinson) and FlowJo (Tree Star).

### Statistical analysis

Data were analyzed using GraphPad Prism (version 6.01; GraphPad software). Statistical analysis was performed using Student’s *t* test or Mann–Whitney test, with Bonferroni post tests; Log-rank or Gehan-Brislow-Wilcoxon tests; or two-way ANOVA, as appropriate. Differences were considered significant for *p* < 0.05.

## Results

### Isolation of anti-CD127 antibodies from a synthetic antibody phage-display library

To generate an antibody against the human IL-7Rα subunit (CD127) using phage-display technology, we first biotinylated the lysine residues of a recombinant product corresponding to the extracellular portion of the protein. Because the protein was produced in mammalian cells and thus was heavily glycosylated, the efficiency of biotinylation was confirmed using a band-shift assay, rather than by mass-spectrometry. Incubation of biotinylated extracellular-domain of IL-7Rα with avidin led to the formation of complexes, which are stable in SDS-PAGE, whereas unbiotinylated IL-7Rα did not shift (Supplemental Figure S1).

**Fig. 1:**
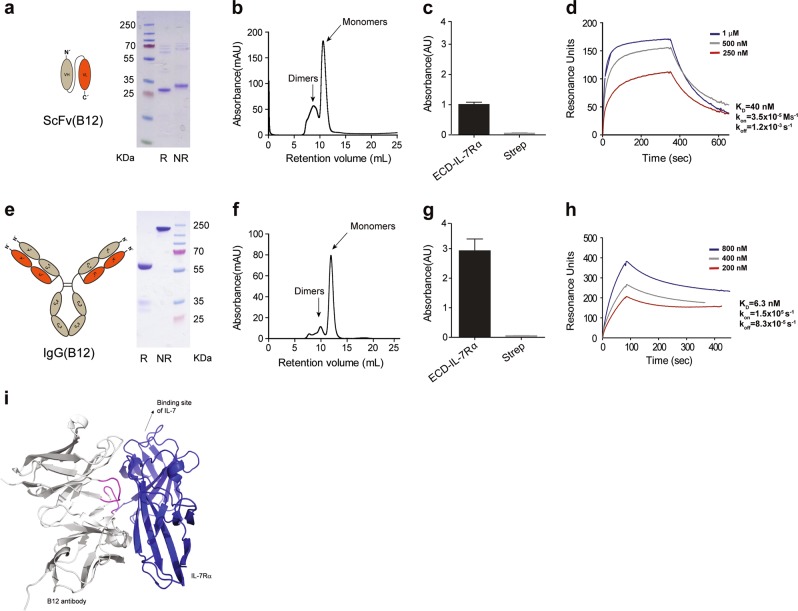
Characterization of the B12 antibody in scFv and IgG format. **a** Schematic domain structure of B12 in scFv format (left), corresponding to a molecular weight of 25 kDa, as shown in SDS-PAGE (right) in both reducing (R) and non-reducing (NR) conditions, and monomeric scFv molecules were separated from dimers by **b** size-exclusion chromatography (S75 column). **c** Specific binding of the antibody to biotinylated-ECD of IL-7Rα is shown by enriched ELISA signal as compared with control streptavidin. **d** Binding kinetics were performed by SPR technology on a BIAcore machine using CM5 microsensor chip. **e** Schematic domain structure of B12 reformatted into IgG (left), expressed in mammalian CHO cells with a molecular mass of 150 kDa (right) and purified by **f** size-exclusion chromatography (S200 column). **g** Improved ELISA signal toward IL-7Rα and **h** increased SPR signal as compared to the scFv **c**, **d**). **i** Representative snapshot from 0.5 μs MD simulations in explicit water. The amino acids that constitute the CDR of the antibody are in magenta. A movie of the MD simulations can be found in the online Supplementary Information

Monoclonal antibodies specific to IL-7Rα, in scFv format, were isolated using both ETH2Gold and PHILODiamond libraries [22, 23]. After three rounds of selection, each of the libraries gave multiple ELISA-positive clones. Most of these clones were further characterized by DNA sequencing, to exclude repetitive clones. We chose the B12 clone (Figure S1) for further analysis, owing to its characteristics in ELISA and binding in a BIAcore-based screening assay. scFv(B12) was purified to homogeneity and analyzed by size-exclusion chromatography, SDS-PAGE, and ELISA, confirming the presence of a protein of the expected size and with IL-7Rα-binding capacity (Fig. 1a–c). The real-time binding kinetics of scFv(B12) towards IL-7Rα immobilized on CM5 chip were analyzed by BIAcore, revealing a dissociation kinetics constant (*K*_d_) of 40.8( ± 17.5) nM, with *k*_on_ = 3.5( ± 1.2) × 10^−5^ M ^−1^ and *k*_off_ = 1.2( ± 0.6) × 10^−3^ s^−1^ (Fig. 1d).

We next reformatted scFv(B12) into full-length human IgG1, using an expression vector for production in CHO cells. Characterization of the purified IgG(B12), henceforth referred to as B12, revealed a lower apparent K_d_ value of 6.311( ± 4.4) nM, with *k*_on _= 1.5( ± 0.56) × 10^5^ M s^−1^ and *k*_off_ = 8.3 ( ± 3.11) × 10^−5^ s^−1^ in BIAcore experiments, owing to the bivalent nature of the antibody (Fig. 1e–h).

### Modeling and MD simulations predict stable B12 binding to human IL-7Rα in a different site from IL-7

Next, we sought to predict the region where B12 binds to IL-7Rα. We first generated a homology model of B12 antibody through the SWISS-MODEL platform [26] and then ran 0.5 µs MD simulations to obtain an equilibrated structure. The amino acids that constitute the CDR of the antibody are all located in the same region of the protein, validating our 3D B12 model. We then conducted docking calculations between the modeled B12 and unglycosylated human IL-7Rα ectodomain (pdb ID: 3DI2) to obtain the structure of the complex, as described in the Methods. Our simulations indicate that the complex is stable, and the binding site is different from that of human IL-7 (Fig. 1i). Although IL-7 is positioned at the elbow region connecting the D1 and D2 domains of IL-7Rα (shown with a black arrow in Fig. 1i) [32], B12 is located on the opposite side. The interface B12/IL-7Rα largely comprises hydrophobic and van der Waals interactions, although a few intermolecular hydrogen bonds exist in the binding interface (Figure S2).

### B12 recognizes both wild-type and human IL-7Rα

**Fig. 2:**
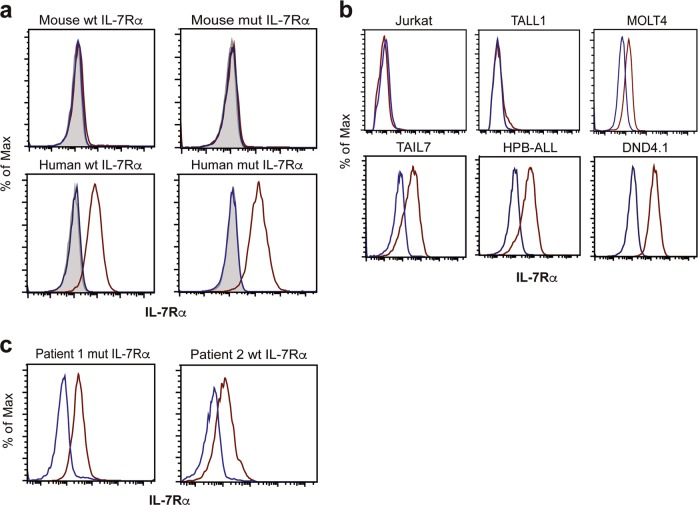
B12 recognizes both the wild-type and gain-of-function mutant forms of the human IL-7Rα. Cells were incubated with 125 mm (18 μg/mL) B12 and an anti-human IgG alexa 647 secondary antibody and analyzed by flow cytometry: **a** Ba/F3 cells transduced with either wild-type (wt) or mutant (mut) versions of the mouse or human IL-7Rα. **b** T-ALL cell lines with different levels of IL-7Rα expression: low/negative (Jurkat, TALL1), intermediate (MOLT4), high (HPB-ALL, IL-7-deprived TAIL7), all of which are IL-7R wild-type; and DND4.1 (high levels of mutant IL-7Rα). **c** Two primary T-ALL patient samples, either mutant (Patient 1) or wild-type (Patient 2). Red histograms: B12, Blue histograms: secondary antibody only; gray-filled histograms: unstained cells

Using Ba/F3 cells ectopically expressing either human or mouse IL-7Rα we found that B12 recognizes specifically the human receptor and does not  cross-react with the mouse (Fig. 2a). Furthermore, B12 recognizes both the wild-type and different mutant forms of the receptor (Table S2), as demonstrated in Ba/F3 cells (Fig. 2a), T-ALL cell lines (Fig. 2b) and diagnostic T-ALL patient samples (Fig. 2c). As expected, B12 showed minimal or no binding to T-ALL cell lines known to be IL-7R-low/negative, such as Jurkat or TALL1 (Fig. 2b). The antibody also recognized IL-7Rα in human thymocytes (Figure S3).

### B12 inhibits both IL-7-dependent and mutant-dependent IL-7R-mediated signaling and induces leukemia cell death

**Fig. 3:**
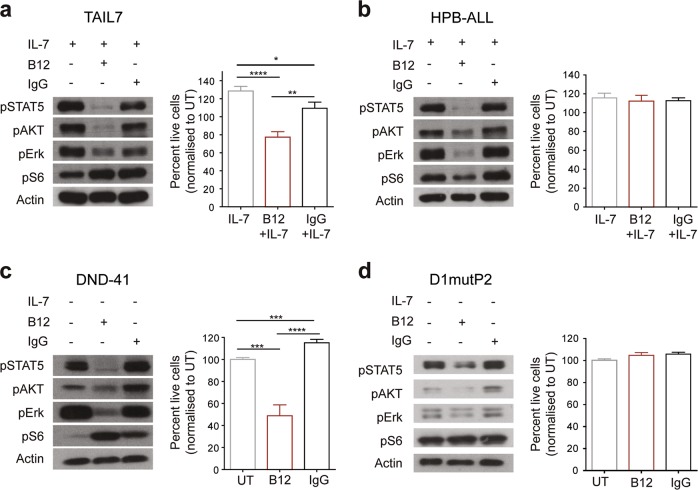
B12 impacts on IL-7/IL-7R-mediated signaling and cell viability. **a** IL-7 starved TAIL7 cells were incubated with 125 nm (18 μg/mL) of B12 and 50 ng/ml of IL-7 together for 120 min at 37 °C. Signaling pathway activation was analyzed by immunoblot using the indicated phospho-specific antibodies (left). Viability was assessed at 120 h (right). **b** Serum starved HPB-ALL cells were treated in a similar manner. Effects on viability were recorded at 120 h. **c** IL-7R mutant DND4.1 cells and **d** D1 cells stably transduced with mutant IL-7Rα (D1mutP2) were treated with B12 and cell survival was analyzed at 120 h. Statistical analysis was performed using two-tailed unpaired *t* test with Welch´s correction (**** *p* < 0.0001, *** *p* < 0.001, ** *p* < 0.01, * *p* < 0.05). Values indicate the mean and SEM of three replicates. In all experiments an irrelevant IgG isotype was used as negative control

Because IL-7/IL-7R-mediated signaling was shown to promote T-ALL cell survival and proliferation in vitro [33] and expansion in vivo [12], we next assessed whether B12 was able to inhibit signal transduction downstream from the receptor and thereby affect cell survival. B12 clearly downregulated IL-7- (Fig. 3a, b) and mutant IL-7R- (Fig. 3c, d) dependent signaling. However, it only induced cell death in some of the cell lines (Fig. 3). This may reflect the lack of IL-7-dependence in HPB-ALL cells (Fig. 3b) or the relatively mild inhibitory effect in D1 cells (Fig. 3d), as opposed to cell lines in which the antibody had a strong negative effect and that are well-known to depend on IL-7 signaling, as is the case of TAIL7 [33], or displaying endogenous *IL-7R* mutation, as is the case of DND41 [12].

### B12 enhances NK-mediated T-ALL cytotoxicity

**Fig. 4:**
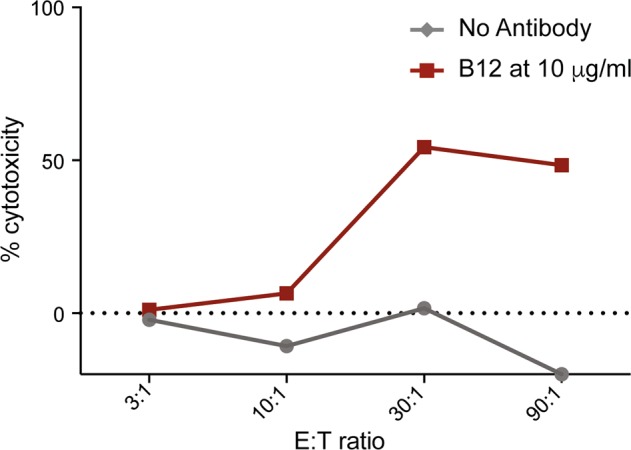
B12 promotes antibody-dependent cell-mediated cytotoxicity in vitro. **a** IL-7R-expressing patient-derived xenograft (PDX) T-ALL-5 cells were cultured with freshly isolated NK cells in the presence or absence of 70 nm (10 μg/mL) of B12. The percentage of cytotoxicity was measured as described in the “Materials and Methods”

To investigate whether IL-7R-expressing cells that were are not sensitive to B12 alone can be targeted by ADCC, we co-cultured NK cells with an IL-7R-expressing PDX T-ALL sample in the presence or absence of B12. We found that B12 had a striking effect in promoting the anti-leukemic activity of NK cells (Fig. 4).

### B12 delays tumor development in vivo

**Fig. 5:**
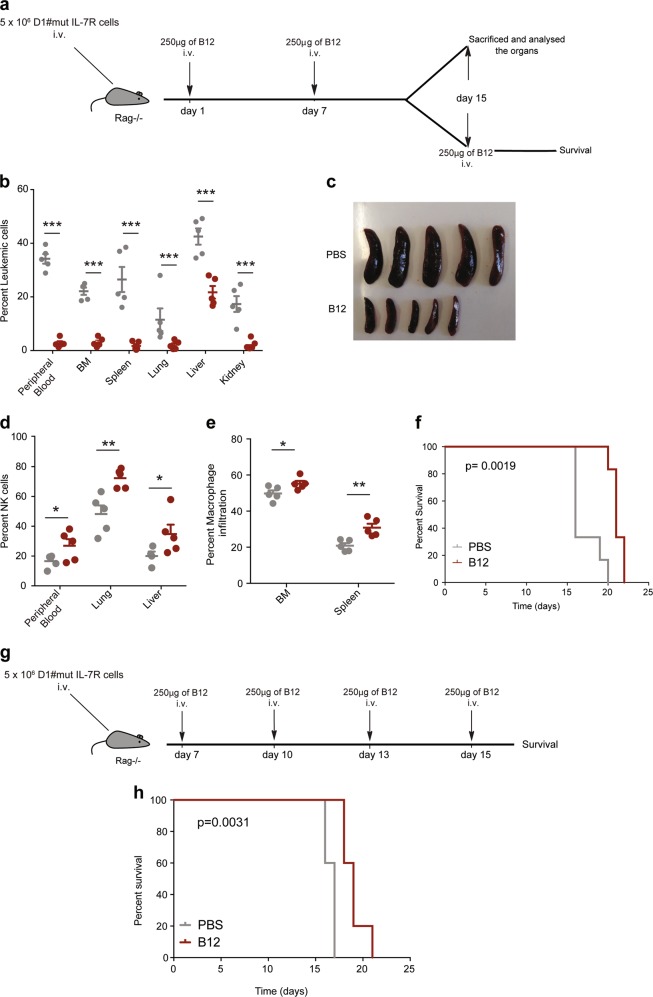
B12 decreases tumor burden and delays T-cell leukemia progression in vivo. **a** Treatment and analysis scheme. Rag1−/− mice (females, *n* = 5 per group) were injected with 5 × 10^6^ D1mutP2 cells via lateral tail vein injection. A day later mice were randomly distributed and injected with 250 μg B12 via the same route. PBS was used as the vehicle control. Injections were administered every 7 days. At day 15, a group of mice were killed and tumor burden in various organs was analyzed, while in another group treatment continued and survival was monitored. **b** Percentage of leukemic cells in the indicated organs was analyzed using flow cytometry, wherein the percentage of GFP-positive leukemia cells within the live cell population was considered as the leukemia burden. **c** Analysis of spleen size. Frequency of **d** NK and **e** macrophage cell infiltration into the indicated organs was analyzed by flow cytometry. Statistical analysis was performed by unpaired *t* test (***p < 0.001, **p < 0.01, *p < 0.05). **f** Kaplan–Meier survival curves of mice treated with vehicle (median survival 16 days) or B12 (median survival 21 days). Statistical analysis was performed using the Log-Rank (Mantel–Cox) test. **g** Experimental scheme for treatment starting at day 7 post-transplantation, when leukemia cells are already clearly detected in the blood. **h** Kaplan–Meier survival curves of mice treated with vehicle (median survival 17 days) or B12 (median survival 19 days). Statistical analysis was performed using the Log-Rank (Mantel–Cox) test

Given the ability of B12 to eliminate leukemia cells in vitro either directly or by promoting the cytotoxic activity of NK cells, we then evaluated the impact of the antibody on leukemia growth in vivo. First, we tested a minimal residual disease-like model of aggressive T-cell leukemia by starting antibody administration 1 day after cell transplantation (Fig. 5a), when leukemia cells are not yet detectable in the blood [34]. At day 15, mice were killed and organ infiltration analyzed. B12 had a major effect on leukemia expansion, strikingly decreasing involvement in the peripheral blood, bone marrow, spleen, lung, kidney, and, to a lesser degree, in the liver (Fig. 5b and S4). In agreement with the diminished frequency of malignant cells in the spleen, B12 reversed splenomegaly (Fig. 5c). As B12 did not kill IL-7R mutant-expressing D1 cells in vitro (Fig. 3d), these effects were likely mediated by an ADCC effect in vivo, possibly via NK cells (Fig. 5d) and/or macrophages (Fig. 5e). Despite the striking differences in organ infiltration at day 15, the disease eventually progressed even in the presence of B12. Nonetheless treatment with the antibody significantly prolonged survival of the mice (Fig. 5f). To test the effect of B12 on full blown leukemia, we started B12 administration at day 7 (Fig. 5g), when D1 cells are easily detected in the blood. Again, B12 significantly prolonged mouse survival (Fig. 5h).

Results in D1 cells were recapitulated using human T-ALL cells. We transplanted HPB-ALL cells subcutaneously into recipient mice and started treatment when tumor masses were clearly detectable. Mice were killed when tumors reached ~ 800 mm^3^. B12 significantly delayed tumor growth, prolonging mouse survival (Figure S5a). We also transplanted a PDX sample intravenously into NOD/SCID mice and started treatment at 6 weeks, when human CD45 + cells were detectable in the blood ( > 3%). Although in this case B12 did not have an effect on survival, it clearly decreased leukemia cell frequency in the blood (Figure S5b) and the relative levels of T-ALL cells negatively correlated with the frequency of NK cells in the blood (Figure S5c).

### B12 cooperates with dexamethasone in inducing leukemia cell death

**Fig. 6:**
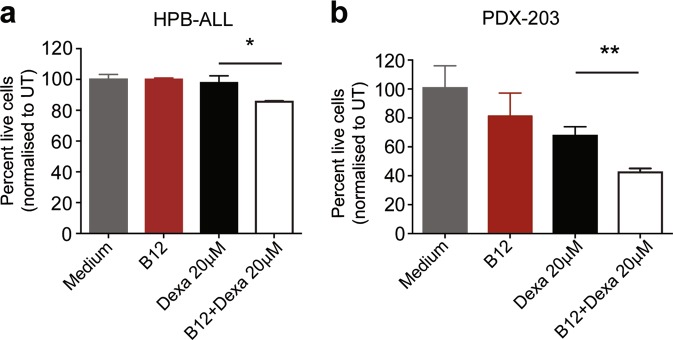
B12 cooperates with dexamethasone in inducing leukemia cell death in vitro. **a** HPB-ALL and **b** PDX 203 cells were cultured in regular medium and treated with the indicated concentration  of dexamethasone and 125 nm (18 μg/ml) B12 for 72 h. Cell viability was determined by Annexin V/7AAD staining and data were analyzed by flow cytometry. Graphics represent the mean and SEM of triplicates normalized to untreated cells. Statistical analysis was done using unpaired *t* test (*p < 0.05, **p < 0.01)

Combined chemotherapy, involving the administration of corticosteroids (dexamethasone or prednisolone) and other drugs, is the mainstay in T-ALL treatment. To have an initial understanding of whether B12 could be potentially integrated into current clinical protocols, we cultured T-ALL cells in the presence of dexamethasone alone or in combination with B12. We found that B12 augmented cell death in combination with dexamethasone both in dexamethasone-resistant HPB-ALL cells (Fig. 6a) and in a dexamethasone-sensitive PDX sample we analyzed (Fig. 6b). These results indicate that B12 may potentiate the effect of corticosteroids in eliminating T-ALL cells and provide proof-of-concept for the combination of anti-IL-7R antibodies with current chemotherapeutic protocols.

### B12 is rapidly internalized and traffics to the lysosome

**Fig. 7:**
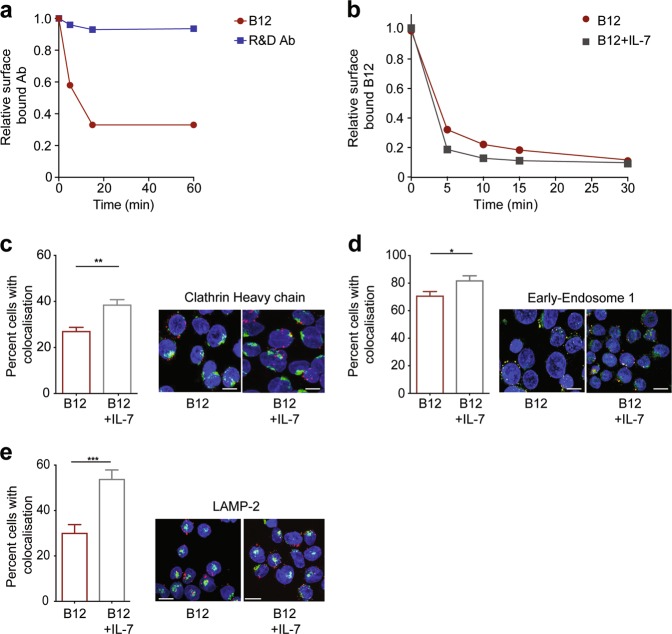
B12 gets rapidly internalized via clathrin-coated pits and traffics into early endosomes and lysosomes. **a** HPB-ALL cells were treated with 125 nm (18 μg/mL) B12, for the indicated time points at 37 °C and surface-bound B12 was analyzed by flow cytometry, as described in “Materials and methods”. Relative signal of B12 bound to IL-7Rα at the cell surface for each time point was calculated as the geometric mean intensity of fluorescence normalized to time 0. A commercially available anti-IL-7Rα from R&D was used for comparison. **b** HPB-ALL cells were cultured in the presence or absence of IL-7 (50 ng/mL) with B12, for the indicated time points. **a**, **b** Data represent the mean from at least two independent experiments. **c** Analysis of B12 colocalization with clathrin was performed by confocal microscopy. HPB-ALL cells were plated on poly-d-lysine coverslips, incubated with B12 at 4 °C for 45 min and subsequently with secondary anti-human IgG Alexa 647 (red). After the incubation with B12, cells were shifted to 37 °C to let the internalization happen. Clathrin-coated pits were detected after 30 min of incubation, by permeabilizing the cells and staining with anti-clathrin-alexa 568 (green) while the nuclei was stained with DAPI. Images were acquired using LSM 880 and the representative picture is a maximum intensity projection of a Z-stack image, 63× objective. For analysis, at least 10 fields of images were analyzed, each image having ~ 100 cells in the frame. Percent of cells with internalization were analyzed by normalizing the cells with colocalized (yellow) spots to the total cells having both the fluorophores. **d** B12 localization in early endosomes was detected after 30 min of incubation at 37 °C, using an anti-EEA-1-alexa 555 antibody, and trafficking to lysosomes after 60 min **e** was detected using an anti-LAMP-2-alexa 488 antibody. Statistical analysis was performed using unpaired *t* test (***p < 0.001, **p < 0.01, *p < 0.05)

Current antibody-based anticancer therapies include the use of antibody–drug conjugates (ADCs). These may be particularly useful in patients whose malignant cells may be refractory to B12 alone and/or in which an ADCC response may be impaired. To understand whether B12 displays appropriate characteristics for cargo intracellular delivery we analyzed B12 internalization and trafficking. We found that, contrary to a commercial anti-IL-7Rα antibody, B12 is rapidly internalized (Fig. 7a), and that IL-7 appears to accelerate B12 internalization (Fig. 7b). Upon internalization, B12 apparently follows the same intracellular route as IL-7Rα [30], trafficking via clathrin-coated pits (Fig. 7c) to the early-endosome (Fig. 7d) and then the lysosome (Fig. 7e). These features suggest that, if engineered into an ADC, B12 should appropriately deliver its cargo into target cells [35].

### B12-mono-methyl auristatin E antibody–drug conjugate kills primary and patient-derived xenograft T-ALL cells more efficiently than naked B12

**Fig. 8:**
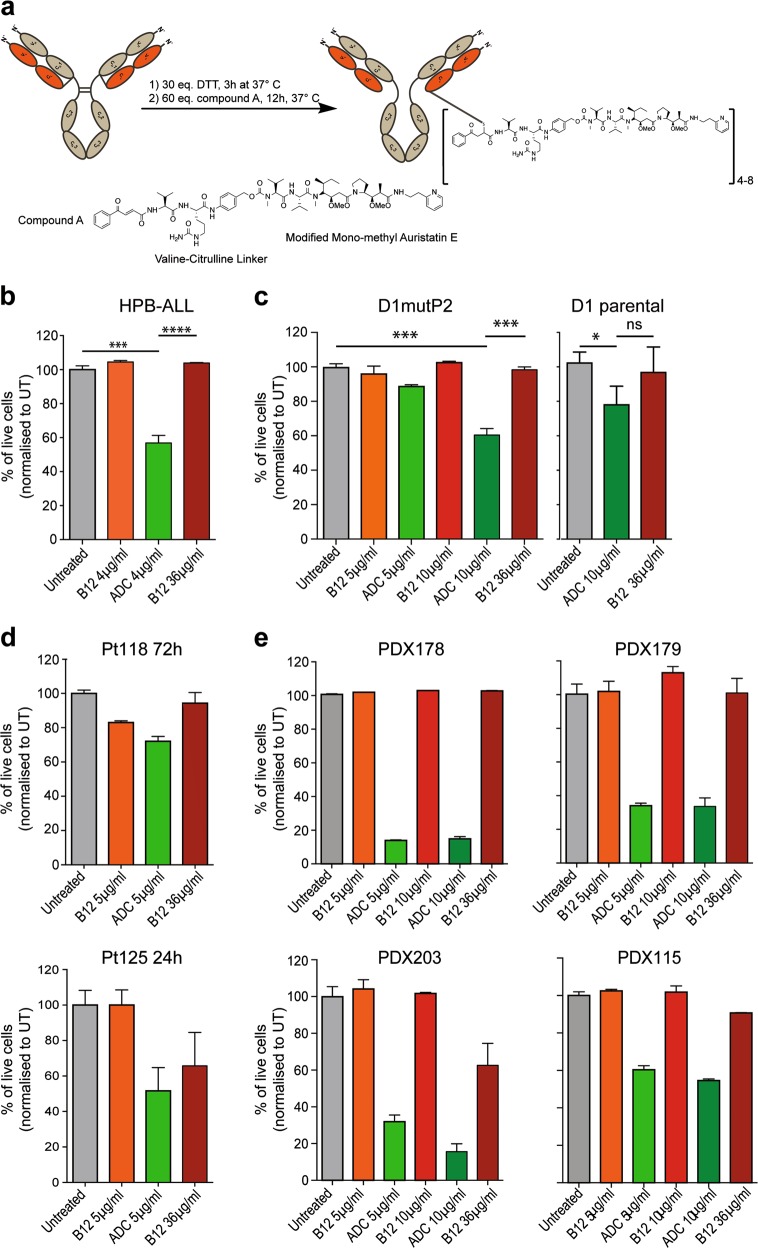
B12–drug conjugate enhances the cytotoxic effect of the native antibody. **a** Antibody in its native form was reduced using 30 equivalents DTT for 3 h at 37 °C in PBS, to reduce the interchain disulfide bonds. The reduced cysteines were then reacted with 60 equivalents of carbonyl acrylic acid–valine-citrulline-monomethyl auristatin E (compound A) for up to 24 h at 37 °C. The average Drug-to-Antibody ratio (DAR) was 4. **b** HPB-ALL cells, **c** D1mutP2 and control D1 parental cells, **d** primary T-ALL cells collected from diagnostic patients, and **e** PDX samples were treated with B12 or B12–drug conjugate at the indicated concentrations. Cell death was analyzed after 72 h of culture, using Annexin V/7AAD staining and data was analyzed by flow cytometry. Percentage of live cells was calculated by normalizing to the untreated cells

Site-specific drug conjugation of B12 was carried out by reducing interchain disulfide bonds and reacting the thiol group of free cysteine with a Michael acceptor (carbonyl acrylic derivate) linked to a cleavable linker (valine–citrulline) and a cytotoxic drug (MMAE) [25]. Using this strategy, we generated the ADC B12–MMAE with an average antibody:drug ratio of ~ 4 (Fig. 8a). B12–MMAE was characterized using native mass-spectrometry wherein the unmodified antibody had a mass of 142,909 Da and, upon conjugation with the drug linker moiety, led to a heterogenous mixture of ADC with mass corresponding to various drug-to-antibody ratios (Figure S6a–c). The ADC retained its binding capacity toward human IL-7Rα (Figure S6d). We then tested B12–MMAE in the cell lines that were refractory to B12 alone in vitro. The ADC significantly promoted cell death in both HPB-ALL and mutant IL-7Rα-expressing D1 cells, contrary to the antibody alone (Fig. 8b, c). Importantly, the B12–MMAE had only a minor impact on parental D1 cells, which do not express the human IL-7Rα (Fig. 8c), indicating that the effects of the ADC owing to unspecific toxicity from MMAE are minimal. We then tested primary T-ALL cells (Fig. 8d) and PDX samples (Fig. 8e), consistently observing a higher effect of the ADC as compared with B12 alone.

## Discussion

Around 70–80% of T-ALL patients present with blasts displaying IL-7Rα and benefit from signals from IL-7 and/or from receptor mutational activation [10, 12, 33]. A clear majority of T-ALL patients may therefore benefit from the introduction of anti-IL-7R therapies into clinical protocols. Signaling-specific small-molecule inhibitors targeting IL-7R-dependent signaling effectors, such as JAK1/JAK3, PI3K/Akt/mTOR, or Bcl-2 may be a valid option [10, 13, 14, 18, 36–41].

Here, we explored alternative, antibody-based strategies to directly target the IL-7R in leukemia cells. There is a rising interest in the potential of anti-IL-7Rα antibodies for treatment of autoimmune, chronic inflammatory diseases, and, more recently, also leukemia [42–54]. However, with few exceptions [55], the studies published so far have essentially reported the testing of anti-mouse antibodies in the context of murine models of disease (e.g., type 1 diabetes, multiple sclerosis, rheumatoid arthritis, colitis, systemic lupus erythematosus, or Sjögren’s syndrome) and thus are clinically inconsequential for humans. In contrast, we generated a fully human antibody, B12, recognizing human IL-7Rα both in the wild-type form and in different gain-of-function mutated variants that drive T-ALL. B12 impairs IL-7R-mediated signaling and is able to promote T-ALL cell death in vitro and delay leukemia progression in vivo, demonstrating obvious pre-clinical value. Consequently, B12 (or further engineered versions of it) can translate directly into clinical applications in T-ALL. Moreover, B12 or other antibodies such as those described in the accompanying paper by Durum and collaborators, can become valid tools in the clinical arsenal against other cancers, especially B-ALL but also chronic lymphocytic leukemia, other hematological malignancies and even solid tumors with ectopic expression of IL-7R, such as breast and lung cancer, where IL-7 and its receptor have been implicated [56–59]. Evidently, these antibodies may as well prove of use in autoimmune and chronic inflammatory conditions.

B12 displays several characteristics that deserve consideration. First, B12 cooperates with dexamethasone in inducing leukemia cell death in vitro, not only in dexamethasone-sensitive T-ALL cells but also in a dexamethasone-resistant cell line. Patients with *IL7R* mutations were recently shown to have very high risk in relapsed disease [11]. This may, in part, relate to the fact that gain-of-function mutations in the IL-7R signaling pathway confer resistance to glucocorticoids [18]. As such, anti-IL-7R antibody-based therapies may be particularly appealing to prevent or manage relapse in T-ALL.

Second, although B12 recognizes IL-7Rα not only in malignant cells but also in normal thymocytes, the effect on thymocyte viability appears to be minimal. The reason for this is not obvious. It may relate to the different ways in which the signaling is wired downstream from the IL-7R in normal versus malignant cells [56]. However, the ability of B12 to induce T-ALL cell death is heterogeneous and only in some cases does B12 kill leukemia cells per se. This happens despite the fact that B12 generally impairs IL-7R-mediated signaling. Intriguingly, we found that the T-ALL cell lines most sensitive to B12 were the ones in which clear signaling inhibition (of STAT5, Akt, and Erk) was paralleled by upregulated phospho-S6. The ability of antibodies to modulate signaling in ways that are not strictly agonistic or antagonistic is of biological and potential clinical importance [60]. These considerations apart, the fact that healthy thymocytes are less affected by antibody treatment than T-ALL cells could be a positive feature in the clinic. Also noteworthy is the fact that hematopoietic stem cells do not express or rely on IL-7R-mediated signaling. Thus, even if particular normal hematopoietic subpopulations are eliminated by the antibody, the system should be able to recover after treatment termination and disease eradication.

Finally, contrary to the antibodies in the accompanying paper, which do not appear to internalize (making them especially adequate for ADCC), B12 showed remarkably fast internalization kinetics, with substantial trafficking into lysosomes. Although B12 also demonstrated the ability to induce ADCC in vitro, and likely in vivo, these features are notably useful for optimal intracellular cargo delivery in ADCs.

Overall, our studies and those of Durum and collaborators, demonstrate the feasibility, and the underlying promise for further developments, of antibody-based strategies targeting IL-7R for the treatment of T-ALL and other pathological conditions in which IL-7 and IL-7R have a role.

## Supplementary information

Supplementary information

Supplementary video
