# Supplementary material for: A fully human anti-IL-7Rα antibody promotes antitumor activity against T-cell acute lymphoblastic leukemia
Source: Leukemia. 2019 Mar 8;33(9):2155–68. doi: 10.1038/s41375-019-0434-8 (PMC6733707; doi:10.1038/s41375-019-0434-8)
Supplement: Supplementary file 1 — Supplementary information [file 41375_2019_434_MOESM1_ESM.pdf]

A fully human anti-IL-7Rα antibody promotes anti-tumor activity against T-cell acute lymphoblastic leukemia

Padma Akkapeddi, Rita Fragoso, Julie A. Hixon, Ana Sofia Ramalho, Mariana L. Oliveira, Tânia Carvalho, Andreas Gloger, Mattia Matasci, Francisco Corzana, Scott K. Durum, Dario Neri, Gonçalo J. L. Bernardes*, João T. Barata*

SUPPLEMENTARY INFORMATION

SUPPLEMENTARY METHODS

Biotinylation of Ectodomain of IL-7Rα / CD127. The extracellular domain of IL-7Rα (CD127-ECD), encoding amino acids Met 1-Gly 236 of human IL-7R (NP_002176.2), was purchased from Life Technologies. The recombinant protein expressed in mammalian cells, contained poly-his tag at the C-terminal. Prior to carrying out Phage Display selections, the protein was biotinylated using EZ-Link Sulfo-NHS-LC-Biotin (Pierce) according to the manufacturer's protocol and purified using PD-10 desalting columns. Since the protein was expressed in mammalian cells and thus harbors heavy glycosylation, the biotinylation was quantified by performing Band-shift assay. In brief, the biotinylated protein was incubated with Avidin and the complex was run on SDS-PAGE, along with respective controls, to observe a shift in the mass due to the interaction between avidin and biotinylated protein.

MD simulations protocol. The MD simulations were performer with AMBER 16 package.(1) The protein (or the complex) was immersed in a water box with a 10 Å buffer of TIP3P(2) water molecules. The system was neutralized by adding explicit counter ions (Na+). All subsequent simulations were performed using the ff14SB force field, which is an evolution of the Stony Brook modification of the Amber 99 force field force field (ff99SB).(3) A two-stage geometry optimization approach was performed. The first stage minimizes only the positions of solvent molecules and ions, and the second stage is an unrestrained minimization of all the atoms in the simulation cell. The systems were then gently heated by incrementing the temperature from 0 to 300 K under a constant pressure of 1 atm and periodic boundary conditions. Harmonic restraints of 30 kcal·mol–1 were applied to the solute, and the Andersen temperature coupling scheme (4) was used to control and equalize the temperature. The time step was kept at 1 fs during the heating stages, allowing potential inhomogeneities to self-adjust. Water molecules are treated with the SHAKE algorithm such that the angle between the hydrogen atoms is kept fixed. Long-range

electrostatic effects are modelled using the particle-mesh-Ewald method.(5) An 8 Å cutoff was applied to Lennard-Jones and electrostatic interactions. Each system was equilibrated for 2 ns with a 2 fs time step at a constant volume and temperature of 300 K. Production trajectories were then run for additional 0.5 μs under the same simulation conditions.

Flow cytometry analyses in human thymocytes. Binding of Ig(B12) antibody to native IL-7Rα expressed at the surface of primary thymocytes was analyzed by flow cytometry. In brief, frozen thymocytes were thawed and subjected to a gradient centrifugation using Ficol. Live cells were collected and incubated with B12 at 4°C for 30min. Cells were then washed with ice-cold PBS and the primary antibody was detected using a commercial Goat alexa 647-conjugated anti-human (H+L) antibody (ThermoFischer Scientific). While CD4 and CD8 populations were identified by using anti-human CD4-FITC and anti-human CD8-PerCP (eBiosciences) antibodies. Cell viability under different experimental conditions was determined by forward scatter/side scatter distribution. Samples were acquired using FACS Fortessa I/II (BD Bioscience) and analyzed using FlowJo (Tree Star).

Histopathology. Mice were sacrificed with anesthetic overdose, necropsies were performed and selected organs (lung, spleen, liver, kidney, brain, spinal cord and femur) were harvested, fixed in 10% neutral-buffered formalin, embedded in paraffin and 4μm sections were stained with hematoxylin and eosin (H&E). Bones were further decalcified in Calci-Clear™ (Fisher Scientific) prior to embedding. Tissue sections were examined by a pathologist blinded to experimental groups, in a Leica DM2500 microscope coupled to a Leica MC170 HD microscope camera. Organ/tissue infiltration and expansion by leukemia cells was scored using a 6-tier system with 0-5 grading scale: 0, absent (no leukemia cells);

1, minimal (rare leukemia cells); 2, mild (<10% infiltration); 3, moderate (10-50% infiltration); 4, marked (50-80% infiltration); 5, extreme (organ almost completely infiltrated by leukemia cells).

Antibody therapy and characterization studies in subcutaneous and PDX mouse models. HPB-ALL ($5x10^6$) cells were injected subcutaneously into Rag1-/- mice. Tumor growth was measured every other day and the treatment was started as soon as the tumors were detectable. 250 μg B12/injection was given five times intra peritoneally every other day. Tumor growth was monitored and mice were sacrificed when the tumor size reached ~$800mm^3$. In another model, patient derived xenograft cells (PDX 203) were engrafted systemically via lateral tail vein injection (10^7 cells) into NOD/SCID mice. Treatment started at 6 weeks post engraftment, when human CD45-positive leukemia cells were detectable in peripheral blood (> 3%). Antibody was administered weekly, five times in total, at a concentration of 250 μg/injection. Leukemia cells were detected by staining for human CD45 (eBiosciences), whereas NK cells were detected by staining with anti-CD56 NK antibody (eBiosciences). Organs were collected for post mortem analysis by flow cytometry and histology.

SUPPLEMENTARY TABLES

Table S1. Lowest energy solution found with PatchDock Server and FireDock.

Structure #	Etotal	aVdW	rVdW	ACE	inside	aElec	rElec	laElec	lrElec	HB	piS	catpiS	catpiS 2	aliph
4	-63.23	-24.03	13.03	-12.57	18.6	0	0	0	0	-2.83	-6.5	0	0	-2
6	-33.5	-22.02	11.67	-3.25	22.14	0	0	0	0	-0.23	-2	0	0	0
10	-32.36	-27.66	12.49	10.12	17.74	-40.76	18	0	7.39	-7.52	-5.5	-0.5	-0.5	0
9	-28.2	-31.76	14.14	12.23	13.58	0	0	0	1.82	-4.43	-3.5	-1.5	-1.5	0
8	-16.44	-6.6	1.78	2.99	15.76	-39.71	0	0	0	-2.12	-1.5	0	0	0
3	-0.85	-1.77	0.31	1.22	13.09	0	0	0	0	-0.28	0	0	0	0
5	0.29	-2.63	0.71	3.92	15.14	-16.5	4.73	-5.4	3.6	0	0	0	0	0
1	1.29	-2.22	0.99	2.52	11.96	0	0	0	0	0	0	0	0	0
7	9.25	-13.37	50.17	1.79	17.55	0	0	0	0	-1.81	-1.5	0	0	0
2	90.37	-36.69	228.71	8.56	14.75	-3.83	13.13	-2.54	10.59	-8.8	-2	-1.5	-1.5	0

Structure # - Solution Number; Etotal - Global Energy, the binding energy of the solution; aVdW, rVdW - softened attractive and repulsive van der Waals energy; ACE - atomic contact energy (ACE); inside - insideless measure; aElec, rElec - attractive and repulsive short-range Coulomb electrostatics; laElec, lrElec - attractive and repulsive long-range Coulomb electrostatics; HB - hydrogen and disulfide bonds; piS - PI-PI stacking; catpiS - cation-PI stacking; aliph - aliphatic interactions

Table S2. IL-7Rα sequences in cell lines and primary T-ALL patient samples

Cell line/ patient samples	IL-7Rα	Sequence of IL-7Rα
**DND4.1**	Mutant	PEINNSSGEMDPILLSRCLTINILSFFSVALLVILACVLWKK
**D1mutP2**	Mutant	PEINNSSGEMDPILLTCPTISILSFFSVALLVILACVLWKK
**Patient 1**	Mutant	PEINNSSGEMDSILNPCLTISILSFFSVALLVILACVLWKK
**Patient 2***	Wild type	PEINNSSGEMDPILLTISILSFFSVALLVILACVLWKK

*Patient 2 is wild type and is shown for reference. All other cell lines used in the study that are not listed here are also wild type. Mutations (insertions or compound insertions plus single aminoacid substitutions) are highlighted in red.

SUPPLEMENTARY FIGURES

a

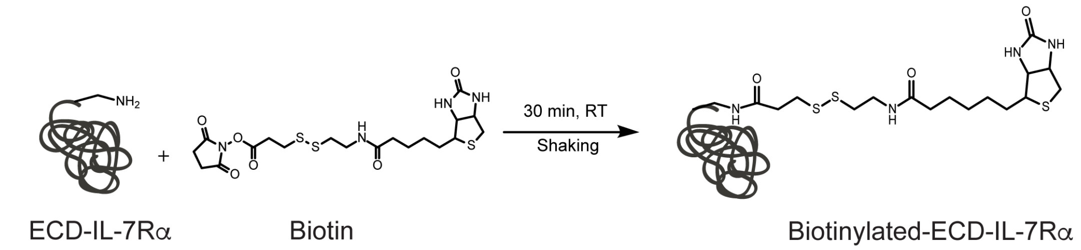

b

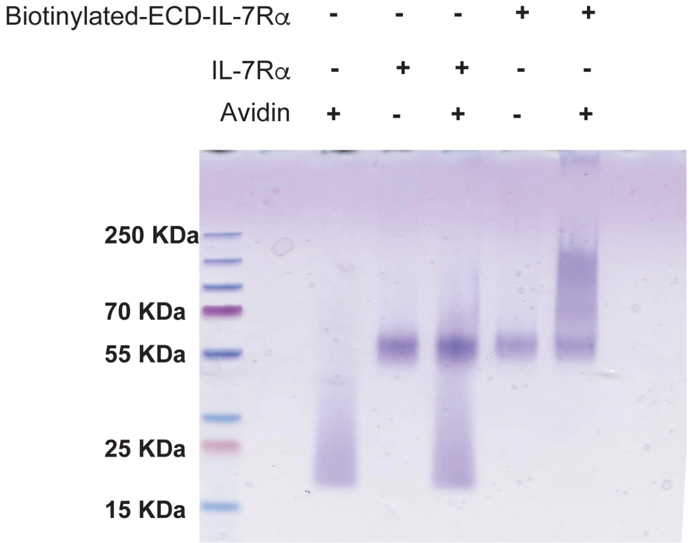

c *Leader peptide* ——— *VH* ——— *Linker* ——— *VL* ——— **amber** ——— **Myc tag**

d MKYLLPTAAAGLLLLAAQPAMAEVQLLESGGGLVQPGGSLRLSCAASGFTFSSYAMSWVRQA
PGKGLEWVSAISGSGGSTYYADSVKGRFTISRDNSKNTLYLQMNSLRAEDTAVYYCAKWVSLP
TFDYWGQGTLVTVSSGGGGSGGGGSGGGGSSELTQDPAVSVALGQTVRITCQGDSLRSYYA
SWYQQKPGQAPVLVIYGKNNRPSGIPDRFSGSSSGNTASLTITGAQAEDEADYYCNSSDVHMP
YVVFGG GTKLTVLGAAAEQKLISEEDLNGAA*

Figure S1. Biotinylation of extracellular domain of IL-7Rα and phage display library overview. (a) Reaction scheme of ECD-IL-7Rα biotinylation. **(b)** Band-Shift assay showing the biotinylation profile of ECD-IL-7Rα. A significant shift in the biotinylated ECD-IL7Rα (~50 KDa) band (extreme right lane) can be seen upon complexation with avidin protein (16.4 KDa). **(c)** Structural details of ETH2 Gold scFv phage display library **(d)** Sequence of the antibody B12.

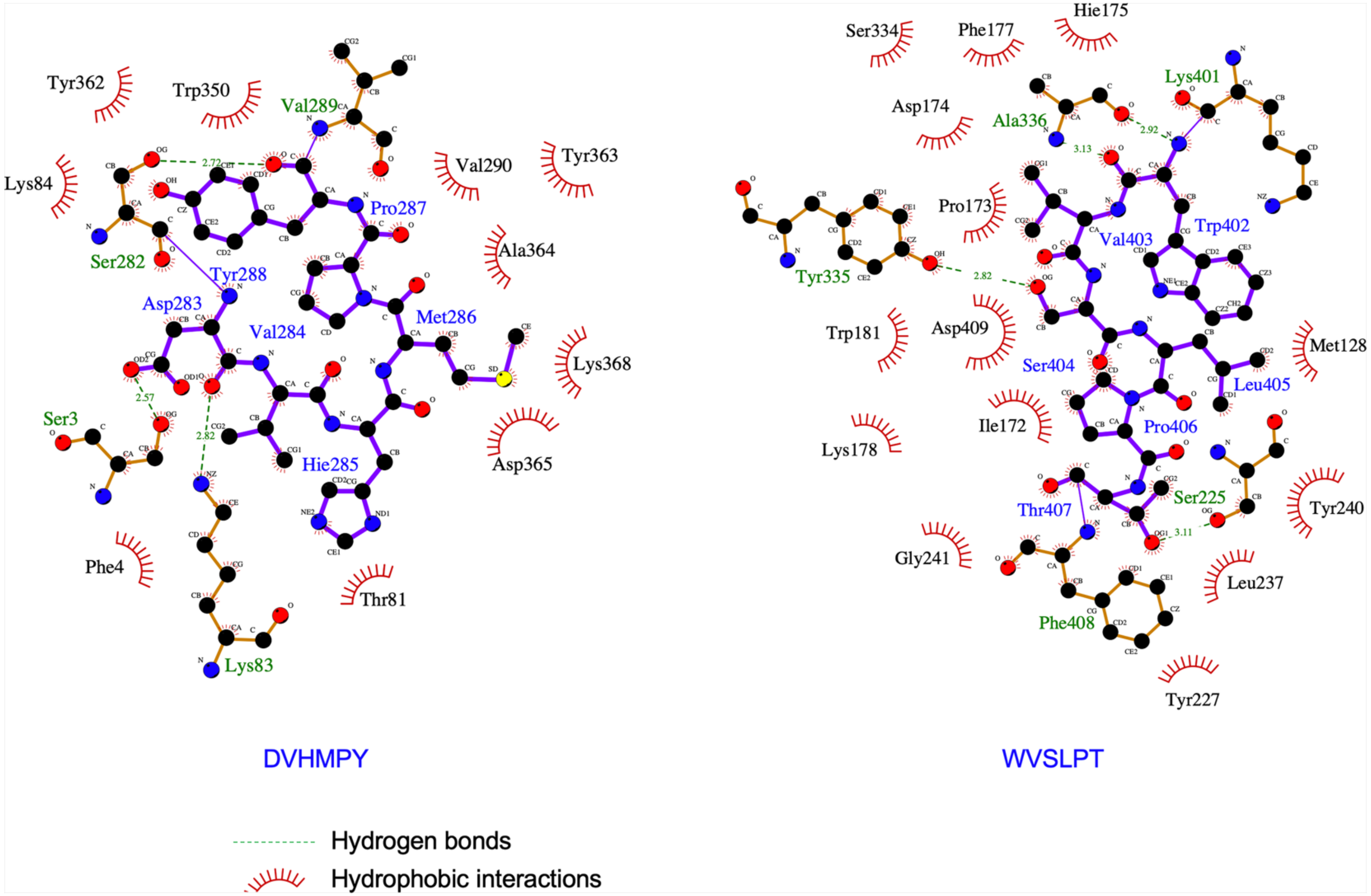

Figure S2. 2D antibody B12/IL7-Rα diagram obtained with *LigPlot+* software (6) for a representative snapshot taken from 0.5 μs MD simulations in explicit water. The residues of the CDRs of B12 are shown in blue.

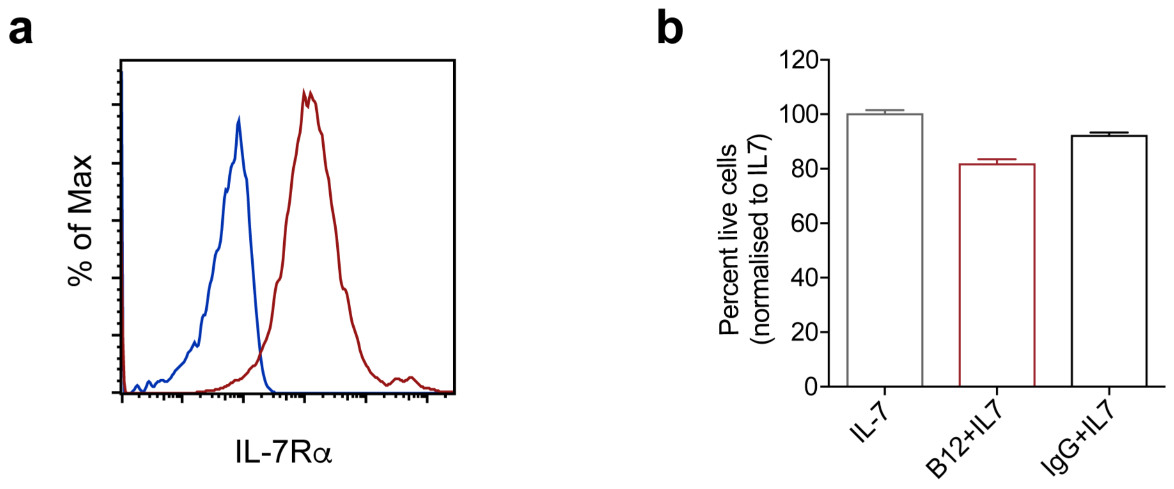

Figure S3. B12 recognizes IL-7Rα expressed on human primary thymocytes but does not have a major impact on their viability. (a) Binding of B12 to IL-7Rα expressed on primary bulk thymocytes was detected by flow cytometry. B12 detects IL-7Rα in the different CD4/CD4 thymocyte subpopulations (not shown). **(b)** Representative graph of effect of B12 on the viability of thymocytes cultured also in the presence of IL-7 for 72h. Mean and SEM of triplicates of two independent experiments are shown.

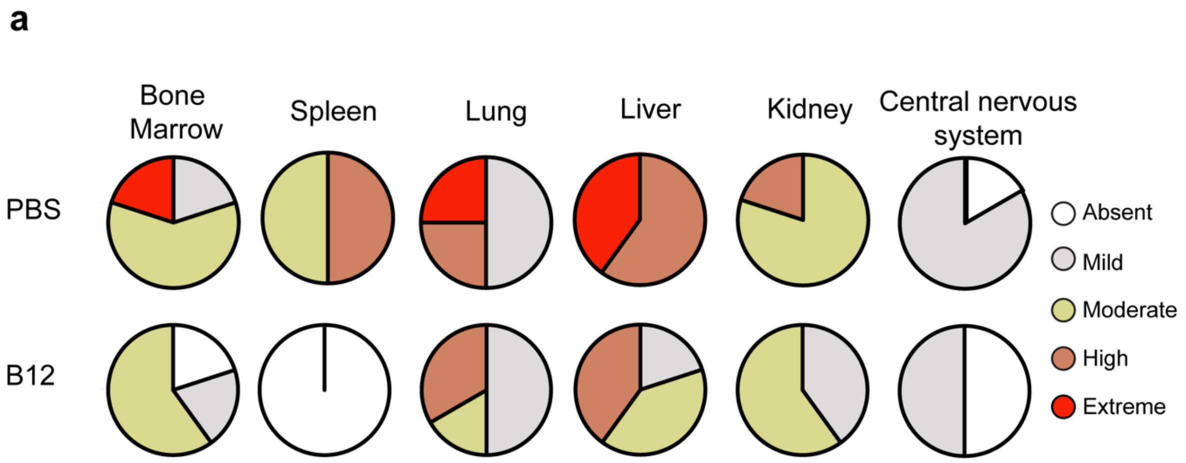

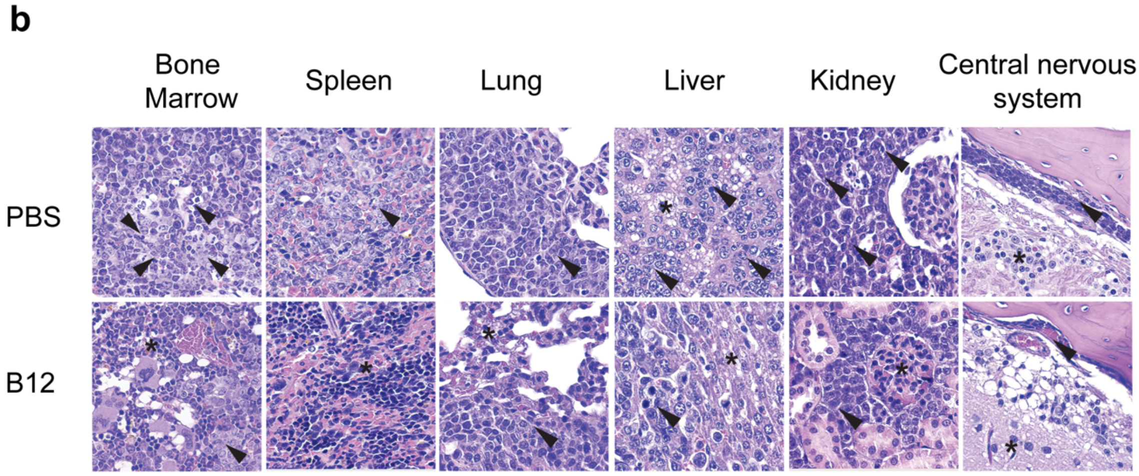

Figure S4. Tumor cell expansion and infiltration in organs of mice xenotransplanted with D1mutP2 cells untreated or treated with B12. **(a)** Pie charts showing lower severity of multiorgan metastatic dissemination in mice treated with B12. Organ/tissue infiltration by leukemia cells was scored using 6-tier system with 0-5 grading scale. **(b)** Representative microphotographs of leukemia cell expansion in hematopoietic organs and of infiltration/metastatic dissemination in secondary target organs (**arrowhead**, tumor cells; **asterisk**, organ parenchyma). Hematoxylin and Eosin: original magnification 400x.

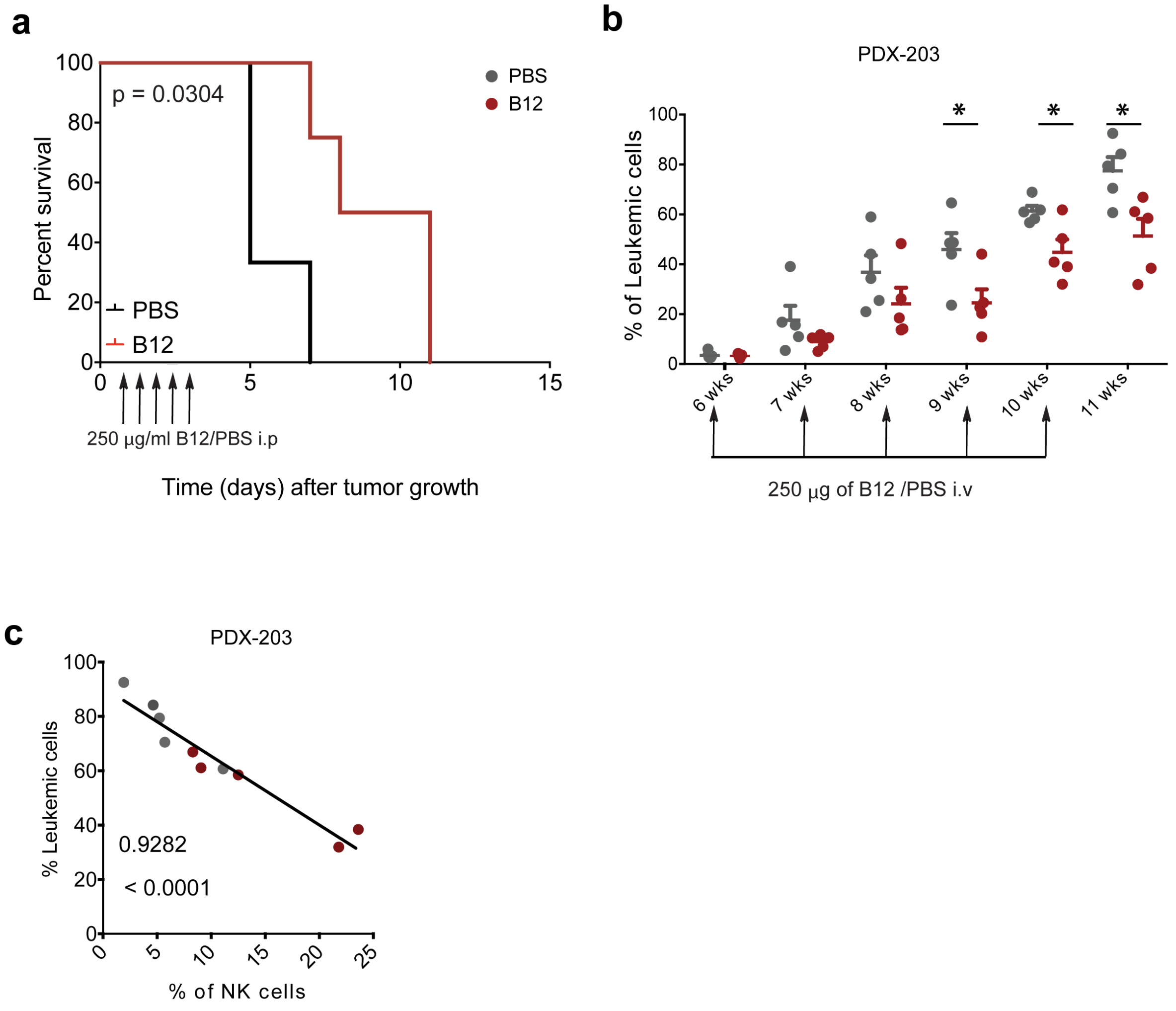

Figure S5. B12 promotes tumor regression in mouse models of human T-ALL. (a) Kaplan-Meyer survival curves of B12-treated (median survival 11 days) and vehicle control-treated (median survival 6 days) Rag-/- mice (females, n=5, per group) subcutaneously implanted with HPB-ALL cells. Statistical analysis was performed using the Gehan-Breslow-Wilcoxon test. **(b)** Percentage of leukemic cells upon treatment with B12, in NOD-SCID mice engrafted with 10 x 10^6 PDX203 cells intravenously (i.v) (females, n= 5 per group). The percentage of human CD45-positive leukemia cells within the live cell population was considered as the leukemia burden. Statistical analysis was performed using unpaired t-test; * $p < 0.05$. **(c)** Negative correlation between leukemia burden and NK cell frequency in peripheral blood, at week 11. Statistical analysis was performed using linear regression; $p < 0.0001$ and $R^2 = 0.9282$.

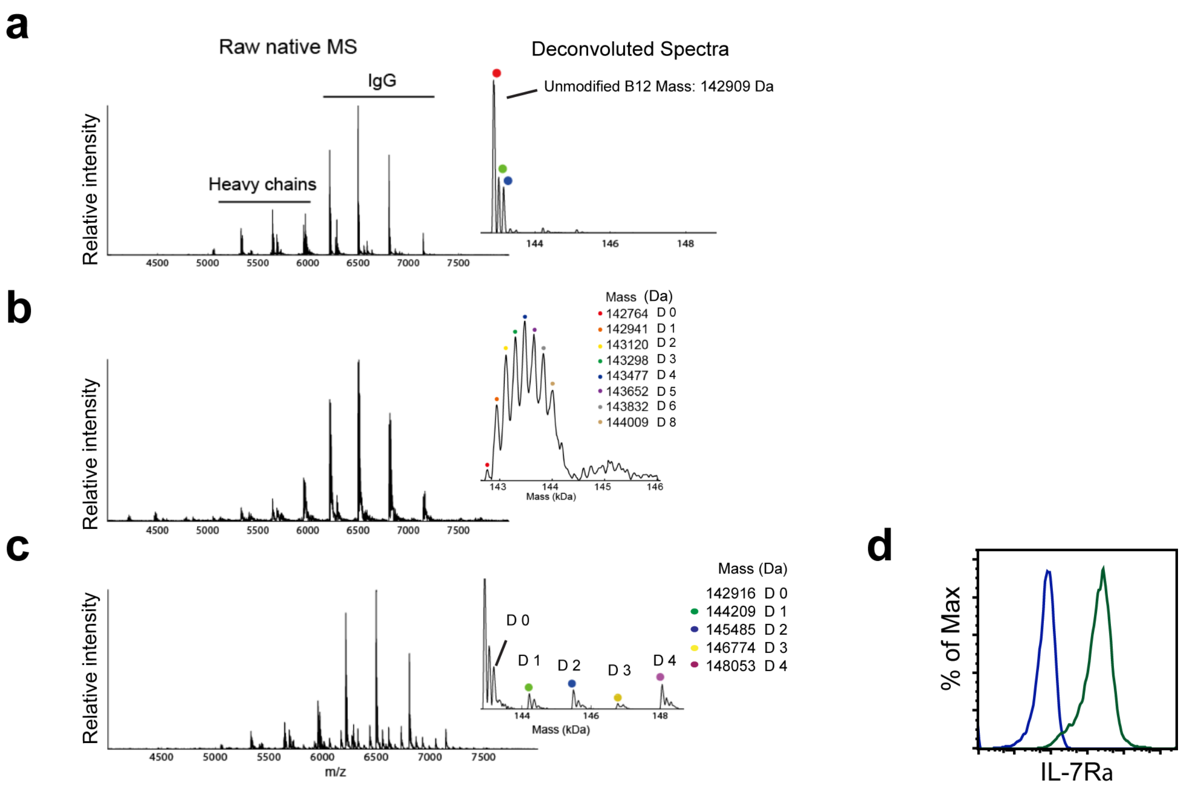

Figure S6. Characterization of B12 drug conjugate (a) Native mass spectrometry of unmodified B12 with average mass being 142909 Da. Shown are both raw native MS and deconvoluted spectra (right). **(b)** Reaction conditions were optimized using the head group carbonyl acrylic acid and the approximate average Drug-to-Antibody ratio DAR was determined using native mass-spectrometry. **(c)** Spectra showing the B12-CAA-val-cit-MMAE wherein the DAR distribution varies from 0 to 4 and the average DAR was assumed to be 4. **(d)** B12-MMAE drug conjugate retains the antigen recognition ability as seen by flow cytometry on HPB-ALL cells. Green histogram: B12-MMAE plus secondary antibody; Blue histogram: secondary antibody only.

Supplementary references

1. D.A. Case, D.S. Cerutti, T.E. Cheatham, III, T.A. Darden, R.E. Duke, T.J. Giese, H. Gohlke, A.W. Goetz, D. Greene, N. Homeyer, S. Izadi, A. Kovalenko, T.S. Lee, S. LeGrand, P. Li, C. Lin, J. Liu, T. Luchko, R. Luo, D. Mermelstein, K.M. Merz, G. Monard, H. DMY and PAK. Amber 2017 [Internet]. University of California, San Francisco. 2017. p. 1. Available from: http://ambermd.org/

2. Jorgensen WL, Chandrasekhar J, Madura JD, Impey RW, Klein ML. Comparison of simple potential functions for simulating liquid water. J Chem Phys. 1983;79:926–35.

3. Viktor Hornak, Robert Abel, Asim Okur, Bentley Strockbine, Adrian Roitberg and CS. Comparison of Multiple Amber Force Fields and Development of Improved Protein Backbone Parameters. PROTEINS Struct Funct Bioinforma. 2006;65:712–25.

4. Andrea TA, Swope WC, Andersen HC. The role of long ranged forces in determining the structure and properties of liquid water. J Chem Phys. 1983;79:4576–84.

5. Darden T, York D, Pedersen L. Particle mesh Ewald: An N·log(N) method for Ewald sums in large systems. J Chem Phys. 1993;98:10089–92.

6. Laskowski RA, Swindells MB. LigPlot+: Multiple ligand-protein interaction diagrams for drug discovery. J Chem Inf Model. 2011;51:2778–86.
